# Crosstalk between sugarcane and a plant-growth promoting *Burkholderia* species

**DOI:** 10.1038/srep37389

**Published:** 2016-11-21

**Authors:** Chanyarat Paungfoo-Lonhienne, Thierry G. A. Lonhienne, Yun Kit Yeoh, Bogdan C. Donose, Richard I. Webb, Jeremy Parsons, Webber Liao, Evgeny Sagulenko, Prakash Lakshmanan, Philip Hugenholtz, Susanne Schmidt, Mark A. Ragan

**Affiliations:** 1 School of Agriculture and Food Science, The University of Queensland, St Lucia, QLD 4072, Australia; 2 Institute for Molecular Bioscience, The University of Queensland, St Lucia, QLD 4072, Australia; 3 School of Chemistry and Molecular Biosciences, The University of Queensland, St Lucia, QLD 4072, Australia; 4 Australian Centre for Ecogenomics, The University of Queensland, St Lucia, QLD 4072, Australia; 5 Advanced Water Management Centre and Centre for Microbial Electrosynthesis, The University of Queensland, St Lucia, QLD 4072, Australia; 6 Centre for Microscopy and Microanalysis, The University of Queensland, St Lucia, QLD 4072, Australia; 7 QFAB Bioinformatics, The University of Queensland, St Lucia, QLD 4072, Australia; 8 Bioinformatics Resource EMBL Australia, The University of Queensland, St Lucia, QLD 4072, Australia; 9 Sustainable Organic Solutions Pty Ltd, Indooroopilly, QLD 4068, Australia; 10 Sugar Research Australia, Indooroopilly, QLD 4068, Australia; 11 Australian Research Council Centre of Excellence in Bioinformatics, The University of Queensland, St. Lucia, QLD 4072, Australia

## Abstract

Bacterial species in the plant-beneficial-environmental clade of *Burkholderia* represent a substantial component of rhizosphere microbes in many plant species. To better understand the molecular mechanisms of the interaction, we combined functional studies with high-resolution dual transcriptome analysis of sugarcane and root-associated diazotrophic *Burkholderia* strain Q208. We show that *Burkholderia* Q208 forms a biofilm at the root surface and suppresses the virulence factors that typically trigger immune response in plants. Up-regulation of *bd*-type cytochromes in *Burkholderia* Q208 suggests an increased energy production and creates the microaerobic conditions suitable for BNF. In this environment, a series of metabolic pathways are activated in *Burkholderia* Q208 implicated in oxalotrophy, microaerobic respiration, and formation of PHB granules, enabling energy production under microaerobic conditions. In the plant, genes involved in hypoxia survival are up-regulated and through increased ethylene production, larger aerenchyma is produced in roots which in turn facilitates diffusion of oxygen within the cortex. The detected changes in gene expression, physiology and morphology in the partnership are evidence of a sophisticated interplay between sugarcane and a plant-growth promoting *Burkholderia* species that advance our understanding of the mutually beneficial processes occurring in the rhizosphere.

*Burkholderia* is a versatile, widely distributed bacterial genus with complex taxonomy whose members occupy diverse ecological niches including soil, water, plants and animals^1^. *Burkholderia* species are currently classified into two groups, pathogenic species belonging to the *Burkholderia cepacia* clade (BCC) and plant-beneficial species in the environmental clade (PBE)^2^,^3^. PBE *Burkholderia* have a complex and diverse host niche and are considered as one of the most potent plant-growth-promoting rhizobacteria (PGPR)^1^,^2^ that are ubiquitous in soil and often present in the rhizosphere^2^,^4^, with heavy colonization reported for at least 30 plant species^4^.

PBE *Burkholderia* are common rhizosphere constituents of major agricultural crops including maize, rice, sugarcane, wheat, tomato and potato^5^,^6^,^7^,^8^,^9^,^10^,^11^,^12^,^13^,^14^. A valuable feature of some PGPR *Burkholderia* species is their capacity for biological nitrogen fixation (BNF)^2^,^12^, siderophore production, inorganic phosphates solubilization, indole-acetic acid production and phytopathogen inhibition^15^,^16^.

Overall, PGPR are recognized for their potential to contribute to sustainable cropping systems but the efficacy of PGPR differs widely, with benefits ranging from none to considerable across crops and environmental conditions. This variability is attributed to environmental factors and difficult-to-predict but specific host interactions^17^,^18^. Considering that the molecular mechanisms of the interaction of PGPR *Burkholderia* with their host plants are largely unknown, knowledge of these interactions will aid in advancing effective microbial inoculants for sustainable crop systems.

Previously, we isolated and characterized a new PGPR *Burkholderia* species (strain Q208 named after Australian sugarcane variety Q208) from the sugarcane rhizosphere and demonstrated its nitrogen-fixing activity in gnotobiotic culture^19^. The *Burkholderia* species heavily colonizes roots and stimulates growth of sugarcane plantlets^19^. Here, we advance our previous research to apply a dual transcriptomic analysis and investigate the interplay between *Burkholderia* Q208 and sugarcane plants. We demonstrate that the association consists of sophisticated mechanisms that enable the mutual adaptation characteristic of a beneficial association between plant and bacterium.

## Materials and Methods

### Plant growth conditions

Plantlets of the Australian commercial sugarcane cultivar Q208 were micro-propagated under sterile conditions on Petri dishes containing half-strength Murashige and Skoog (MS) medium^20^. Media were supplemented with 1% sucrose, adjusted to pH 5.5 and solidified with 0.3% phytagel (PhytoTechnologies, Kansas USA). Plant cultures were maintained at 28 °C and 16/8 h light/dark cycle with photon flux density 400 μmol m^−2^ s^−1^ during the light cycle. Uniform and fully developed plantlets with ≈2–3 cm roots were used for bacterial inoculation experiments.

### Bacterial inoculation of plants

*Burkholderia* Q208 and *Bacillus megaterium* (used as a control in oxygen saturation and Confocal Raman microscopy measurements) were grown to mid-log phase in nutrient broth medium (Sigma-Aldrich), centrifuged at 4,500 × g for 15 min, washed twice and resuspended in sterile water. Bacterial solutions at an absorbance at 600 nm of ≈1 (approximately 10^9^ cell per ml) were used for inoculations. Roots of plantlets were dipped in bacterial solution for 15 min before transfer into sterile 500-ml containers containing 200 ml of half-strength MS medium supplemented with 2% sucrose, 0.3% phytagel and pH adjusted to 5.7. Plants were incubated in a growth cabinet (28 °C, 16/8 h day/night, 400 μmol m^−2^ s^−1^) for 18 days (or as indicated within the text) after inoculation. Non-inoculated plants served as control. Each container contained one plantlet, and each treatment consisted of 10 replicate containers. The containers were randomly rearranged in the growth cabinet daily.

### Biofilm formation assay

A 50-μl aliquot of overnight-grown *Burkholderia* Q208 was inoculated in 5 ml nutrient broth, and incubated at 28 °C with shaking at 200 rpm. After 1 h, 100-μl aliquots were placed in 16 wells of a polystyrene 96-well plate. The first 8 wells received an additional 50 μl sterile phosphate-buffered saline (PBS), while the other 8 wells received an additional 50 μl sterile sugarcane root extract. The root extract was prepared by macerating 2 g of axenic sugarcane root in 5 ml sterile PBS. The plate was incubated overnight at 28 °C with shaking. Assays for biofilm formation were performed using 0.1% crystal violet as previously described^21^.

### Analysis of root morphology and element composition

The morphology of 15 roots (five plants, three primary roots per plant) was analyzed by hand cross-sectioning into ≈15 mm segments at the middle of the root using a vibratome (Leica VT 1200 S, Germany) and observed under confocal laser scanning microscopy. For elemental analysis plants were separated into roots and shoots, rinsed and cleaned three times in 0.5 mM CaCl_2_ to remove traces of growth medium from surfaces. Tissues were dried at 60 °C for five days, weighed, homogenized and analyzed for elemental content. Nitrogen and carbon were determined by combustion (TruSpec CHN analyzer, LECO, MI). Results represent averages of 10 plants. All growth experiments were repeated three times.

### Statistical analyzes

Statistical analyses were performed using Student’s *t* test (GraphPad Prism4, San Diego) to test for significant differences in dry weight and elemental content of inoculated *versus* non-inoculated plants.

### Oxygen (O_2_) saturation measurement

Oxygen saturation in the growth medium was determined using a Dissolved O_2_ meter SG6 (SevenGo^TM^ Mettler Toledo, Schwerzenbach) following the manufacturer’s instructions.

### Confocal laser scanning microscopy (CLSM)

Centre sections of primary roots (~15 mm long) were washed, embedded in 3% agarose and sectioned with a vibratome (Leica VT 1200 S, Germany). Roots were coated with agarose before processing to ensure that bacteria on the root surface were trapped in the agarose and not dislodged during cutting. Sections were transferred into curved slides, washed thoroughly with deionized water and analyzed by CLSM.

### Transmission electron microscopy (TEM)

Five roots were fixed in 2.5% glutaraldehyde in 0.1 M phosphate buffer pH 6.8 overnight at 4 °C. After washing in 0.1 M phosphate buffer, roots were postfixed in 1% osmium tetroxide, dehydrated through a graded ethanol series, infiltrated with Epon and polymerized for 2 days at 60 °C. Thin sections were cut with a Leica Ultracut UC6 ultramicrotome, picked up on carbon-coated copper grids, stained with uranyl acetate and Reynold’s lead citrate^22^ and viewed in a JEOL 1010 TEM at 80 kV. Images were captured on an Olympus Soft Imaging Solutions Megaview III digital camera.

### Scanning electron microscopy (SEM)

Plant roots fixed in 2.5% glutaraldehyde in 0.1 M phosphate buffer were dehydrated in a graded ethanol series and dried in a Tousimis Autosamdri-815 critical point dryer. Roots were then sputter-coated with platinum and viewed in a JEOL 7001 FEGSEM at 5 kV.

### Confocal Raman microscopy

A loop of freshly grown bacterial cells was spread on optically polished CaF_2_ slides (Edmund Optics Singapore). Raman spectra were acquired using an Alpha 300 Raman/AFM (WITec GmbH, Ulm) equipped with a 100x N.A. 0.9 objective (Nikon)^23^. A frequency-doubled continuous-wave Nd:YAG laser stabilized at 532 nm was used for excitation. Laser power was kept below 5 mW at the sample. Raman signals were collected with a 100-μm optical fiber with resolution 4 cm^−1^. Spectral acquisition was performed at integration time 0.2 s, which proved to be sufficient for high-contrast resonance spectra for cytochromes. All images were constructed by collecting spectra on 80 points/line with 80 lines per/image at scan span 30 μm.

### Quantification of bacteria by RT-qPCR

Gnotobiotic plantlets inoculated with *Burkholderia* Q208 (1.25 × 10^9^ CFU/ml) were grown for 1, 5 or 15 days. Roots were washed twice with MgSol (10 mM MgSO_4_) and separated into non-sterilized or surface-sterilized roots. Surface sterilization was modified after Govindarajan *et al*.^24^. Briefly, roots were washed with MgSol and vortexed for 3 min under agitation in 2% sodium hypochlorite. Roots were rinsed five times for 5 min in sterile H_2_O, then rolled onto Luria-Bertani agar plates to verify surface sterility. Total *Burkholderia* Q208 dsDNA was extracted using MoBio PowerSoil DNA isolation kits following the manufacturer’s instructions (MoBio Laboratories, Carlsbad CA). *Burkholderia* 16S rRNA was amplified using *Burkholderia*-specific primers Cluster11F (5′-GAACAGAGGGTTGCCAAG-3′) and 1392R (5′-ACGGGCGGTGTGTAC-3′). Sugarcane 25S rRNA was amplified using the 25S rRNA primer pair 5′-ATAACCGCATCAGGTCTCCAAG-3′ and 5′-CCTCAGAGCCAATCCTTTTCC-3′ 25. RT-qPCR followed the protocol of Paungfoo-Lonhienne *et al*.^26^. After amplification, melting curve analysis was performed to verify uniqueness of the product. Measured Ct values were converted to relative copy-numbers by normalizing the Ct values of bacterial 16S rDNA by the Ct values of sugarcane 25S rDNA. All experiments were performed in three biological replicates, and all measurements in three technical replicates.

### RNA extraction and sequencing

**Figure 1:**
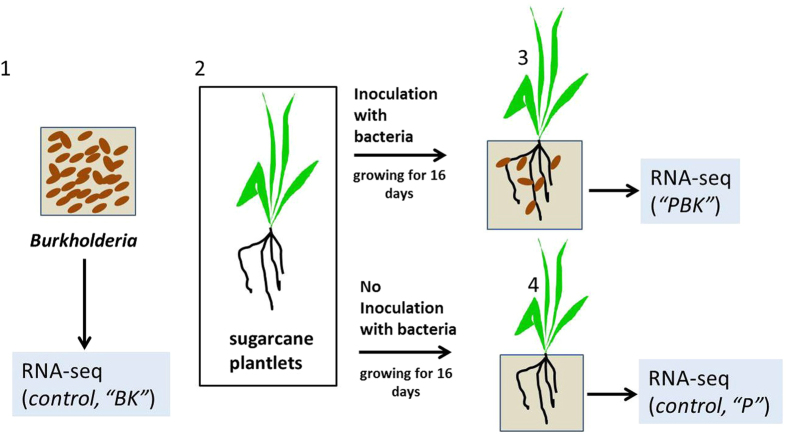
Schematic presentation of the “dual transcriptome analysis”. Sugarcane plantlets (2) were inoculated with *Burkholderia* Q208 culture (1) and plants were grown for 16 days (3). The control, non-inoculated plants (4) were also grown for 16 days. Subsequently, bacterial culture (1), roots of the control plants (4; boxed area), and roots of the inoculated plants (3, boxed area) were subjected to transcriptomic analyses. In the case of (3) the analyses included the transcriptomes of both bacteria and plant roots (“dual transcriptomics”).

Sugarcane plantlets obtained by micropropagation (3 months) were grown in gnotobiotic culture inoculated with *Burkholderia* Q208 (termed ‘PBK’) for 16 days. Controls were *Burkholderia* Q208 grown in the absence of plantlet (bacteria only ‘BK’, inoculated into MS medium using sterile 1 mL pipette) and sugarcane plantlets cultivated in the absence of bacteria (plant only ‘P’) (Fig. 1). Each treatment consisted of three biological replicates. All samples were frozen in liquid N_2_ immediately after collection and stored at −80 °C. Total RNA of samples was extracted using MoBio PowerPlant RNA isolation kits following the manufacturer’s instructions (MoBio Laboratories). Samples were subsequently treated with RTS™ DNase (Applied Biosystems). RNA quantity and quality were verified using a Bioanalyzer (Agilent). Ribosomal RNA of plants and bacteria was removed using a Ribo-Zero Magnetic Kit (plant seeds/root) and a Ribo-Zero rRNA Removal Kit (meta-bacteria) respectively, following the manufacturer’s instructions (Epicentre, Madison WI). Construction of TruSeq RNA libraries and HiSeq2000 sequencing was conducted at the Ramaciotti Centre for Genomics (University of New South Wales, Australia). Libraries were prepared as described in the TruSeq RNAseq v2 Library Preparation guide (Illumina, September 2012) except that rRNA-depleted RNA was input from the “Elute, Prime, Frag” stage of the library preparation. A 12-cycle PCR was used to amplify the adapter ligated library. RNA-Seq libraries were sequenced (independently for each biological replicate) on a 100-bp paired-end lane using the Illumina platform (Illumina HiSeq 2000, San Diego).

### Pre-processing of Illumina reads

All primary sequencing data were deposited in the European Nucleotide Archive (ENA) under accession number ERS568745. All reads were evaluated for quality using FASTQC (http://www.bioinformatics.babraham.ac.uk/projects/fastqc/).

### Mapping of Illumina reads

All RNA reads of BK and PBK libraries were aligned against the *Burkholderia* Q208 genome (Accession number SRP029967) using the Subread V1.4.3 subread-align command. Read pairs aligned to the *Burkholderia* Q208 genome were then counted relative to their unpaired match position against *Burkholderia* Q208 genome features. Reads mapping to two features at the same position were ignored. Sugarcane reads in PBK libraries not mapping to *Burkholderia* Q208 or sugarcane libraries were independently mapped onto the sorghum genome using Bowtie2 version 2.06. Reads which mapped onto sorghum were counted using Subread featureCounts. The *Sorghum bicolor* genome used as reference was Release 21 from ftp.ensemblgenomes.org.

### Quantification of gene expression

All reads from the pure BK *vs* PBK libraries, and from PBK *vs* P libraries, were feature-mapped onto the *Burkholderia* Q208 and *S. bicolor* genomes respectively using Subread featureCount, and the counts were input into DESeq for differential expression analysis. DESeq^27^ is available as a Bioconductor package from the Bioconductor repository^28^ and from http://www-huber.embl.de/users/anders/DESeq. Transcripts mapping to the *Burkholderia* Q208 genome, and those found to be significantly down- or up-regulated by more than twofold (dispersion-corrected mean of biological replicates) with an adjusted P value (*P* < 0.01) were analyzed further. Volcano plots were made in R (www.r-project.org). Functional and Gene Ontology annotations were mapped to the most differentially expressed genes using Blast2GO^29^ available at https://www.blast2go.com.

## Results

### RNA-seq analyses of *Burkholderia* Q208-sugarcane association

In our previous research (Paungfoo-Lonhienne *et al*.^19^), we demonstrated that *Burkholderia* Q208 is a PGPR of Australian sugarcane that abundantly colonizes the roots of host plants, which suggested that the beneficial association occurs through biofilm formation. Thus here, to understand the features underlying the association, we investigated the molecular interplay between the *Burkholderia* Q208 biofilm and the sugarcane roots. To simultaneously capture the molecular mechanisms involved in the establishment of the beneficial association between *Burkholderia* Q208 and sugarcane, we carried out a “dual transcriptomic analysis” to assess the changes in gene expression profiles in both roots and root-associated bacteria (Fig. 1 and Supplementary Figure S1.).

Approximately 6 million read pairs were sequenced from each biological replicate of axenically cultured *Burkholderia* Q208 (‘BK’), and 30 million read pairs from each biological replicate of axenic sugarcane roots (‘P’) and *Burkholderia* Q208 inoculated roots (‘PBK’). Reads were generally of high quality (FASTQC quality scores >28) and there were no concerns regarding the utility of any library. A screen against typical sequencing xenocontaminant DNA revealed no significant contamination for any library.

A full genome sequence of sugarcane is not available, so RNA-seq reads of sugarcane were compared to chromosomal and expressed sequence tags of *Sorghum bicolor*, sugarcane’s closest relative for which the entire genome is publicly available. It has been reported that sorghum and sugarcane genomes are mostly collinear and share 95.2% of sequence identity^30^. Of ≈215 million sugarcane reads (‘P’), approximately 65 million mapped to annotated sorghum features. Of ≈200 million mixed sugarcane + *Burkholderia* RNA reads (‘PBK’) after *Burkholderia* Q208 genomic subtraction, approximately 63 million mapped to sorghum (Table 1).

**Figure 2:**
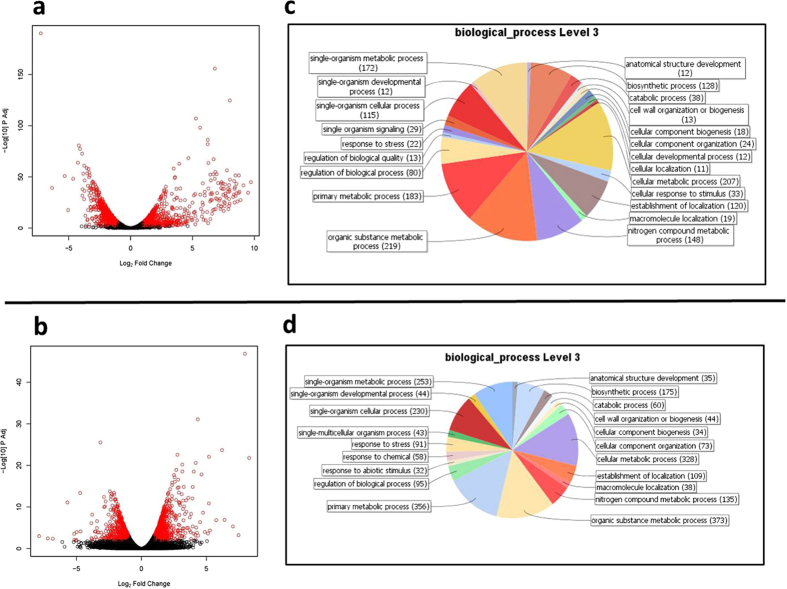
Results of the “dual transcriptome analyses”. (**a)** Volcano scatter plot of fold change (log2) Vs DESeq adjusted P-values (−log10) of *Burkholderia* vs *Burkholderia* colonized sugarcane roots (**a**) or of sugarcane vs sugarcane colonized by *Burkholderia* (**b**). Red circles represent adjusted P values < 0.01 and >2 fold change. Level 3 Gene Ontology (GO) terms distribution of 1,628 unigenes induced in *Burkholderia* colonized on sugarcane roots (**c**) or of 2,606 unigenes induced in sugarcane roots colonized by *Burkholderia* (**d**).

Volcano scatter plots (Fig. 2a,b) summarize the fold-change and statistical significance distributions for transcripts of sugarcane and *Burkholderia* Q208. The plots confirm that the RNA libraries were of high quality. We noticed that many up- or down-regulated genes localize adjacent to each other in the genome of *Burkholderia* Q208. Gene Ontology (Biological Process) annotations show that for both organisms, over half of the most differentially induced genes are involved in metabolic processes: 57% of the bacterial genes (Fig. 2c) and 56% of the plant genes (Fig. 2d) have Level 3 annotation^29^ as Single-organism, Primary, Organic substance, Cellular or Nitrogen metabolic processes. By contrast, genes annotated as stress- or defense-related represent a minor proportion of induced genes with 1.4% and 3.5% for *Burkholderia* and sugarcane, respectively.

### Genes involved in formation of the biofilm are upregulated in *Burkholderia*

**Figure 3:**
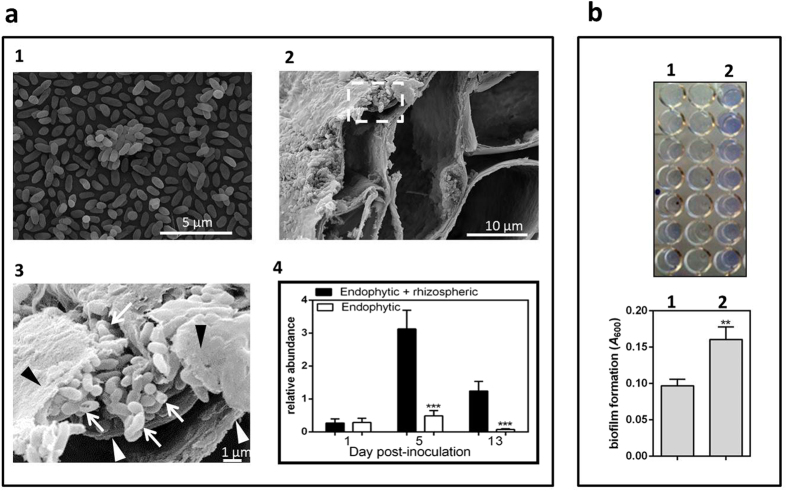
Association of *Burkholderia* Q208 with sugarcane roots. (**a**) Electron microscopy of the sugarcane roots inoculated with *Burkholderia* Q208 and the abundance of *Burkholderia* on the root surface, compared to the endophytic bacteria. a1 – TEM of the bacterial culture used for the inoculation; a2 – SEM of the sugarcane roots with a box indicating the magnified area shown in a3. The plants were grown in gnotobiotic conditions for 5 days. Arrows indicate bacteria, white arrowheads-plant cell wall, black arrowheads-blanket-like material covering *Burkholderia* cells. a4 –quantitative real-time PCR showing relative abundance of *Burkholderia* outside of the roots (rhizospheric) and inside of the roots (endophytic). Black bars display the results of the qRT PCR carried out on the roots with bacteria, and white bars the roots only, with root-associated bacteria stripped off. Abundance of 16S rRNA genes of *Burkholderia* was quantified relatively to 25S rRNA genes of sugarcane. Data represent averages of three independent replicates. *** indicates significant difference at *P* < 0.001 (Student’s *t* test). (**b**) Biofilm formation assay for the conditions when *Burkholderia* were grown in the nutrient medium without (1) or with (2) addition of sugarcane root extract. Blue color indicates formation of the biofilm, when crystal violet staining is applied to the media (top panel); the absorbance quantification of 7 biological replicates are shown (bottom panel). The bars represent SD. ** indicates significant difference at *P* < 0.01 (Student’s *t* test).

**Figure 4:**
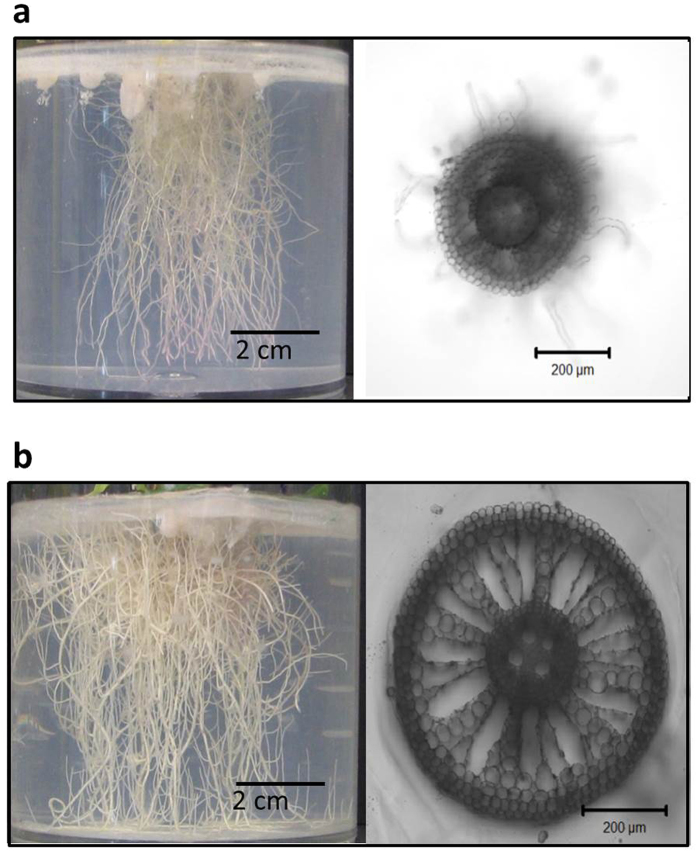
*Burkholderia* Q208 induces the plant root thickening and expansion of the aerenchyma. Sugarcane roots of the plants not inoculated with *Burkholderia* (**a**), and those of the plants inoculated with *Burkholderia* Q208 (**b**). The right panels in (a) and (b) show CLSM images of the sections representing the average thicknesses of the main roots.

A common feature of biofilms is a developed bacterial layer^31^, which was observed when *Burkholderia* Q208 cells are grown in the presence of roots (Fig. 3a,2,3), but not when grown without plants (Fig. 3a,1). Scanning electron microscopy (SEM) analyses established that most of the bacteria are associated with the root surface rather than growing endophytically (Fig. 3a,2). This observation was confirmed by qRT-PCR (Fig. 3a,4), when we determined the abundance of *Burkholderia* Q208 16S rRNA relative to 25S rRNA of sugarcane roots grown with or without microbes. The presence of *Burkholderia* Q208 was considerably higher (5.3 fold (5 dpi) to 43.3 fold (13 dpi) (*P* < 0.001)) in the rhizosphere than in roots, implying that the association is primarily rhizospheric.

We also tested the influence of sugarcane root extract on the ability of *Burkholderia* Q208 to form a biofilm, using the crystal violet assay^21^. Crystal violet is a basic dye which binds to negatively charged molecules, including cell surface and exopolysaccharides (EPS). Bacteria grown in nutrient medium with addition of sugarcane root extract exhibited dense staining with crystal violet (Fig. 3b), implying that sugarcane extract induces formation of the biofilm.

Bacterial biofilms consist of a number of components, among which are EPS, DNA and proteins^32^. The transcriptomic data reveal that the gene clusters *bce-I* and *bce-II* (*Burkholderia cepacia* complex exopolysaccharide-I and –II) directing EPS “cepacian” biosynthesis in *Burkholderia* species^33^ are over-expressed (on average 9-fold, Supplementary Table S1) in *Burkholderia* Q208 associated with sugarcane (Plant-associated *Burkholderia* (PBK) gene expression compared to *Burkholderia* alone (BK), Fig. 1). Similar to *Burkholderia xenovorans*^33^, the genes of clusters *bce-I* and *bce-II* of *Burkholderia* Q208 are adjacent on the genome, forming a cluster of 18 genes involved in nucleotide sugar biosynthesis, glycosyl and acyltransferase activities, polymerization and export of saccharides.

Biofilm formation is generally controlled by quorum sensing (QS), a form of chemical communication that coordinates gene expression when the concentration of bacterial cells reaches a certain threshold^34^. Nitrogen-fixing plant-associated *Burkholderia* display a highly conserved quorum sensing system, designated BraI/R, that is encoded by a cluster of three genes (*braI*, *rsaL* and *braR*), the products of which generate and respond to *N*-dodecanoyl-3-oxo-homoserine lactone^35^; this cluster is also present in the genome of *Burkholderia* Q208 (Supplementary Table S1). In the biofilm *Burkholderia braI* is up-regulated (2-fold) whereas, curiously, *rsaL* and *braR* are down-regulated (to 0.35- and 0.45-fold of reference levels, respectively). However, the absolute counts of raw reads of *rsaL* (16,000) and *braR* (15,500) are greater than the mean (700) read number over all expressed genes, suggesting that although these genes are down-regulated, BraI/R quorum sensing by *Burkholderia* Q208 remains active in the sugarcane rhizosphere.

### Lipo-polysaccharides and flagella biosynthesis genes in *Burkholderia* Q208 are down-regulated

When *Burkholderia* Q208 is associated with sugarcane roots, two bacterial gene clusters involved in lipo-polysaccharides (LPS) production are down-regulated (Supplementary Table S2). Cluster 1 is composed of 17 genes including the O-antigen polymerase (0.21-fold; in other words a 5 fold decrease) involved in O-antigen assembly, and a member of the GtrA protein family (0.18-fold). In *Shigella flexneri*, GtrA encoded by bacteriophage is involved in O-antigen modification^36^. The second cluster contains 12 genes, including UTP-glucose-1-phosphate uridyltransferase (0.32-fold) involved in biosynthesis of bacterial polysaccharides (LPS and EPS) and often associated with pathogenesis: in *Streptococcus pneumoniae* it is considered the main virulence factor^37^. Independently of these two clusters, the lipid A-core-O-antigen ligase involved in ligation of O-antigen onto the core region of the lipid A-core block is also down-regulated (0.37-fold). These results suggest that LPS production by *Burkholderia* Q208 is reduced in the biofilm. Accordingly, the immune response to LPS is apparently not induced in roots of sugarcane; nitric oxide (NO) synthase *NOA1* is down-regulated (0.56-fold, Supplementary Table S3), with NO production being a hallmark of the innate immune system induced by PAMP LPS in plants^38^.

Flagella serve as organs of locomotion facilitating bacterial chemotaxis. In biofilms, bacteria are non-motile and this is achieved by functional regulation or inhibition of flagellar gene transcription^39^. Associated with sugarcane roots, the *Burkholderia* Q208 gene cluster containing 50 genes related to chemotaxis and flagellar synthesis is down-regulated (mean 0.3-fold, Supplementary Table S4), suggesting that motility is inhibited through loss of flagella. In sugarcane roots, expression of the genes involved in the signalling pathway of plant immune response to flagellin, which includes receptors *FLS2* and *BAK1* and the mitogen-activated protein kinase (MAPK) cascade^40^, is not significantly altered (Supplementary Table S3). Thus important immunogenic factors are repressed in *Burkholderia* Q208 when associated with sugarcane, in line with the establishment of a mutualistic association. Remarkably, Cluster 2 involved in LPS biosynthesis (Supplementary Table S2) also contains the flagellar transcription activator *flhD* which is strongly down-regulated (0.07-fold), indicating that regulation of flagella and of LPS production are linked, could be a sign of adaptation to the mutualistic association that prevents activation of the immune defense of sugarcane.

### Upregulation of cytochromes in *Burkholderia* Q208

*Burkholderia* species are obligate aerobes and lack fermentation pathways, but they can live in microaerobic conditions. Analysis of the *Burkholderia* Q208 genome shows that its respiratory chain terminates in two branches, with *bo*- and *bd*-type cytochromes as terminal oxidases^41^. *Burkholderia* Q208 genes encoding cytochrome *bd* occur in duplicate, similar to that of *Escherichia coli*, and encode cytochrome *bd*-I (*cydA* and *cydB*) and *bd*-II (*cbdA* and *cbdB*) complexes^42^. These genes are highly induced in the sugarcane rhizosphere (Supplementary Table S5), *cydA* 34-fold, *cydB* 27-fold, *cbdA* 112-fold, *cbdB* 49-fold. *CydX*, a protein essential for assembly and stability of the cytochrome complex^43^, is upregulated 21-fold. Expression of cytochrome *bo* is, however, unchanged. A high level of induction of cytochrome *bd* was confirmed by confocal Raman microscopy (Supplementary Figure S2). Cytochromes produce strong, representative Raman spectra at 532 nm excitation due to resonance of the porphyrin ring^44^. In the sugarcane rhizosphere, *Burkholderia* cells present strong bands at 752, 1130, 1315 and 1585 cm^−1^, a signature of *Geobacter sulfurreducens* renowned for its high electron-transfer activity^23^. Normalised to CH stretching (2938 cm^−1^), the bands corresponding to cytochromes are more intense in *Burkholderia* Q208 than in *Bacillus megaterium* cells used as control (Supplementary Figure S2).

### Upregulation of energy pathways in *Burkholderia* Q208

The high level of cytochrome *bd* production by *Burkholderia* Q208 suggests a high rate of energy generation, as these cytochromes are involved in oxidative phosphorylation in the electron transport chain. Transcriptomic data of *Burkholderia* Q208 were analyzed to explore the nature of energy pathways operating in the rhizosphere. Glycolysis converts C6 monosaccharides to pyruvate, producing ATP and NADH in both aerobic and anaerobic conditions. Full commitment to glycolysis by *Burkholderia* Q208 is indicated by over-expression of phosphoenolpyruvate synthase (*pps,* 204-fold) and fructose phosphokinase (*fpk,* 159-fold: Supplementary Table S5) representing the key regulatory point at which glycolysis enters irreversibility. Pyruvate dehydrogenase, which catalyses the rate-limiting step of the complex that produces acetyl-CoA from pyruvate, is over-expressed threefold. Citrate synthase is over-expressed fourfold, suggesting increased activity of the Krebs cycle, hence production of GTP and NADH. In aerobic conditions, NADH produced by the Krebs cycle is oxidized by cytochromes of the electron transport chain, generating a trans-membrane proton gradient. As the *bd*-type cytochrome is highly over-expressed, this should increase the trans-membrane proton gradient. Consistent with this picture, the F_0_/F_1_ ATP synthase complex responsible for converting the trans-membrane proton gradient into ATP is highly induced (9 genes, average over-expression 126-fold: Supplementary Table S5).

### Activation of additional energy pathways in *Burkholderia* Q208

Two anaerobic pathways are induced: the oxalate catabolism and the arginine-deiminase pathways. The anaerobic oxalate catabolism pathway has been well-studied in *Oxalobacter formigenes*^45^. Oxalate in *O. formigenes* is imported into the cell and activated to oxalyl-CoA by ligation with coenzyme A transferred from formyl-CoA and catalyzed by formyl-CoA transferase (Frc). Oxalyl-CoA is then decarboxylated by oxalate decarboxylase (Oxc) to CO_2_ and formyl-CoA. After transfer of the CoA moiety onto oxalate, formate is exported from the cell, coupled with the import of oxalate through the oxalate/formate antiporter (OxlT), generating a proton-motive force used by F_0_/F_1_ ATP synthase to produce energy^45^. In the *Burkholderia* Q208 genome, the *oxlT* gene is physically separated from *frc* and *oxc,* and appears to belong to a cluster of five genes including the three following genes: an ortholog of the *E. coli* acetyl-CoA:oxalate-CoA transferase *yfdE*, a hypothetical protein with 3D similarity to a proton symporter, and the PHB depolymerase. This cluster is highly overexpressed in *Burkholderia* Q208 associated with sugarcane (mean 328-fold: Supplementary Table S5). However, we observed almost no reads mapping to the *oxlT* transcript, implying that oxalate is not imported in a conventional way. The NAD-dependent formate dehydrogenase complex that catalyzes formate oxidation in aerobic conditions is down-regulated (Supplementary Table S5), suggesting that oxalate catabolism occurs under microaerobic conditions. The other upregulated pathway (anaerobic arginine-deiminase pathway) involves catabolism of arginine to ornithine with production of ATP. Similar to *Rhizobium etli*^46^, the three genes involved in the pathway are clustered (*arcABC*): arginine deiminase (*arcA*), ornithine carbamoyltransferase (*arcB*) and carbamate kinase (*arcC*). Export of residual ornithine from the cell is coupled with import of arginine through the arginine/ornithine symporter (*arcD*). All four genes are highly over-expressed (mean 320-fold: Supplementary Table S5), suggesting that *Burkholderia* Q208 uses this pathway for ATP generation under microaerobic conditions.

### Genes for PHB accumulation and C4-dicarboxylate transport are upregulated in *Burkholderia* Q208

*Burkholderia* in the biofilm accumulate the carbon storage polymer poly-β-hydroxybutyrate (PHB) as evidenced under TEM as electron-transparent droplets in the cytoplasm (Supplementary Figure S3). PHB production commences from acetoacetyl-CoA and involves reduction of acetoacetyl-CoA to hydroxybutyryl-CoA by PhaB or PhbB enzymes that cause oxidation of one molecule of NADH, followed by polymerization by PhaC or PhbC. PHB mobilization occurs in three steps: depolymerization by a PHB depolymerase to hydroxybutyrate, oxidation of hydroxybutyrate by 3-hydroxybutyrate dehydrogenase yielding acetoacetate, and transfer of CoA to acetoacetate by acetoacetate-succinyl-CoA transferase. The genes involved in all PHB cycling steps^47^ are highly upregulated in *Burkholderia* Q208, most strongly the orthologs of *phbB* (720-fold) and *phbC* (236-fold) (Supplementary Table S5).

*dctA* encodes the structural component necessary for C4-dicarboxylate transport, while *dctB* and *dctC* encode positive regulatory elements^48^. Our data show that when *Burkholderia* Q208 are associated with sugarcane roots, the genes for C4-dicarboxylate transport *dctA, dctB* and *dctD* are over-expressed (5-, 1.6- and 1.4-fold respectively, Supplementary Table S6).

### Morphological changes of plant roots in association with *Burkholderia* Q208 biofilm

Considerable structural changes are observed in roots when the association with *Burkholderia* is established, with increased root thickness brought about by additional layers of cortex cells and larger aerenchyma (Fig. 4). Aerenchyma facilitates gas flow through plant tissues^49^ and is an important adaptation to hypoxic conditions in the rhizosphere. Confirming this notion, the concentration of oxygen in the growth medium of sugarcane inoculated with *Burkholderia* was significantly lower than in the control plants (*P* < 0.001 at top and middle, and <0.0001 at the bottom, of the growth vessels) or when inoculated with *Bacillus megaterium* (*P* < 0.05 at the top-middle, <0.001 at the bottom) (Supplementary Figure S4).

### Hypoxia-related genes are upregulated in sugarcane

Plants subjected to hypoxia increased ethylene production, which is responsible for formation of aerenchyma in the root cortex *via* the action of aminocyclopropane-1-carboxylate synthase (ACC synthase) and ACC oxygenase (ACC oxidase 1)^50^. Analysis of the sugarcane transcriptome suggests a similar mechanism: in sugarcane inoculated with *Burkholderia* Q208, ACC synthase *ACS8* and ACC oxidase 1 are over-expressed (4.6 and 10.8-fold respectively: Supplementary Table S7). Hypoxia increases expression of the haemoglobin ortholog *AHB1/GLB1* in sugarcane (16-fold). Similarly, in *Arabidopsis*, hypoxia-induced expression of *AHB1/GLB1* occurs predominantly in roots, and its function is linked to its ability to bind oxygen^51^. Adaptation to hypoxia is further confirmed by increased expression of the APETALA2/ethylene response factors *RAP2* (3-fold) and especially of prolyl 4-hydroxylase alpha (53-fold). In *Arabidopsis*, RAP2.2 is involved in post-translational modification of hypoxia-induced proteins^52^ and prolyl 4-hydroxylase alpha regulates responses to oxygen deficiency^53^.

### Availability of photosynthetic carbon is required for establishment of effective plant-*Burkholderia* association

Sucrose is the main carbohydrate derived from photosynthesis, and its degradation to hexoses in legume nodules is important for delivering photosynthates to symbionts. Sucrose synthases fulfilling this function are also essential for BNF in *Pisum sativum* (*RUG4*)^54^ and *Phaseolus vulgaris* (*PvSSn*)^55^. In sugarcane inoculated with *Burkholderia* Q208, sucrose synthases 1 and 2, orthologs of *PvSSn* and *RUG4*, were over-expressed (1.5- and 3-fold respectively, Supplementary Table S8), suggesting that hexose production is increased in sugarcane roots.

**Figure 5:**
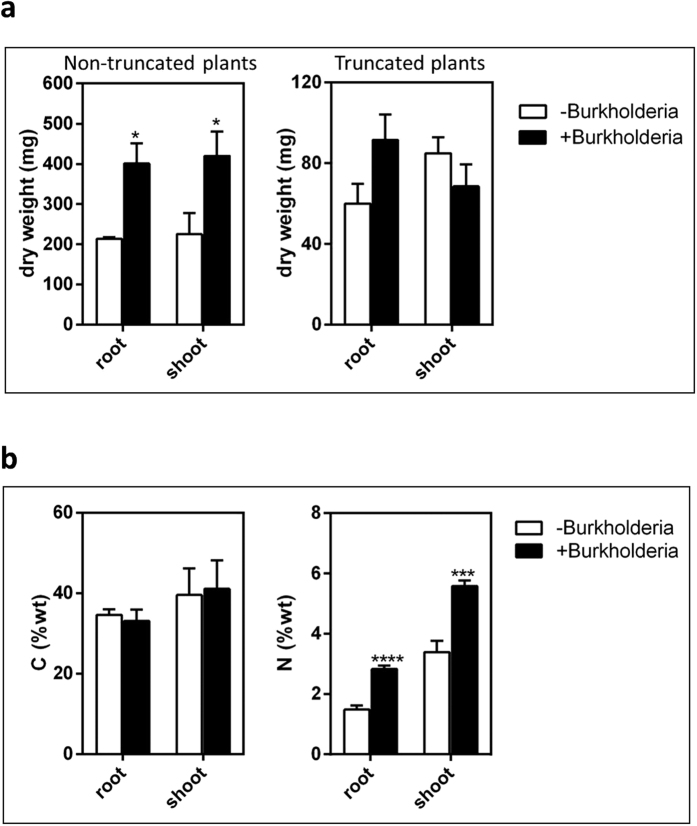
Effect of *Burkholderia* Q208 on growth of sugarcane plants. (**a**) Dry weight of the sugarcane plants, with shoots either not (left panel) or truncated (right panel) at the time of inoculation. Data represent the average of 10 individual plantlets. Asterisks above bars indicate significant differences from the control: *P < 0.05; ***P < 0.001; ****P < 0.0001 (Student’s t test). (**b**) Inoculation with *Burkholderia* Q208 significantly increases nitrogen concentration in the sugarcane plants but does not affect carbon concentration.

To evaluate the dependence of the *Burkholderia* Q208-sugarcane association on plant carbon supply, we truncated sugarcane plantlets before inoculation with the bacterium to reduce the ability of plantlets to provide photosynthates. In truncated plantlets, the effect of *Burkholderia* Q208 on plant biomass was strongly reduced (Fig. 5a), compared to non-truncated plantlets, suggesting that availability of photosynthates is a main driver of the successful relationship. Nevertheless, the role of plant stress in adversely affecting the bacterium-sugarcane association should not be ruled out.

## Discussion

**Figure 6:**
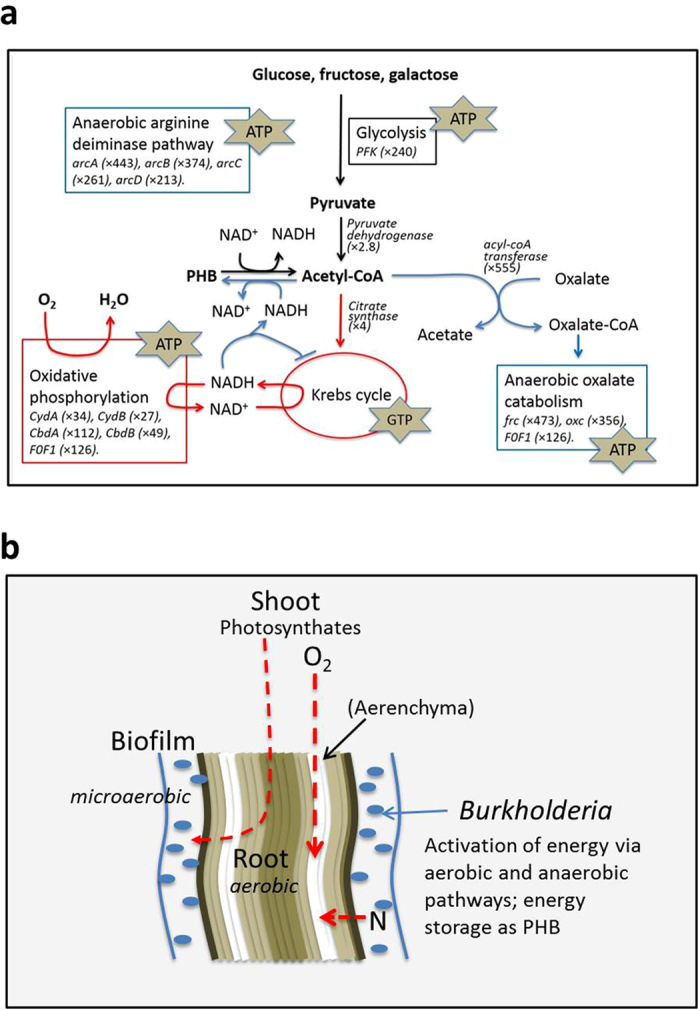
Schematic presentations of *Burkholderia*-sugarcane interactions revealed via “dual transcriptomics” analysis. (**a**) Energy pathways of *Burkholderia* Q208 induced upon association with sugarcane. Arrows and frames indicate energy pathways in aerobic (red arrows) and microaerobic (blue) conditions. All the designated pathways are activated in the biofilm. Stars highlight the energy carriers generated by each pathway and the up-regulation of the particular genes is indicated in the boxes. (**b**) A proposed model for the crosstalk between plant roots and *Burkholderia*. The biofilm is formed by *Burkholderia* cells on the surface of sugarcane roots. Microaerobic conditions are created by the bacteria through activation of the aerobic energy generation (cytochrome *bd* and F0/F1 ATP synthase are over-expressed); this microaeroby presumably supports BNF. The plant provides oxygen to the roots and to the biofilm through enlarged aerenchyma in order to support its own metabolism and that of the bacterial cells. The bacterial energy demand is supported by the plant’s photosynthates, such as malate, succinate and fumarate.

Our morphological analyses reveal that the association between *Burkholderia* Q208 and sugarcane roots is supported by a number of transformations in both partners. We detected significant changes in gene expression in sugarcane and *Burkholderia Q208* by simultaneously analyzing transcriptomes of both partners which match the observed morphological changes. The bacteria form a biofilm on the root surface, lose motility and immunogenicity, activate hexose intake, and generate energy in both aerobic and microaerobic conditions. Plants in turn do not activate their immune response, expand root aerenchyma to conceivably counteract oxygen limitation induced by the bacterial biofilm, and supply the bacteria with photosynthates. In its entirety, the partnership has the hallmarks of a mutually beneficial association that resembles the symbiotic association between rhizobia and legumes at molecular and morphological and molecular levels, resulting in the establishment of a stable bacterial population, and stimulation of plant growth (Fig. 6b).

The apparent first step in the establishment of the association is the formation of a robust bacterial biofilm on roots. Transcriptomic analyses reveal that the flagellar biosynthesis genes in *Burkholderia* Q208 are down-regulated, indicative of a transition of *Burkholderia* from a motile state to non-motile multicellular aggregates, typical for bacterial cells within biofilms^39^.

A PGPR should not be recognized as virulent by the plant and accordingly several key pathways are down-regulated. Two main factors of virulence of pathogenic BCC-clade *Burkholderia* are lipopolysaccharides (LPS) and flagella^56^,^57^. More generally, LPS and flagella are pathogen-associated molecular patterns (PAMPs), responsible for activating the innate immune system of plants^38^,^40^. LPS is the major component of the outer membrane of Gram-negative bacteria^58^, and are formed by three chemically linked components: a hydrophobic glycolipid integral to the membrane (lipid A), a core polysaccharide located at the surface, and a polysaccharide consisting of repeating units of sugars extending from the membrane surface into the environment, known as the O-antigen, as the immuno-dominant portion of the molecule^59^. O-antigen plays also a crucial role in root colonization, as the O-antigen mutants of *Pseudomonas fluorescens* lose this ability^60^,^61^. Among the strongly downregulated genes implicated in LPS biosynthesis in *Burkholderia* Q208 are those involved in O-antigen assembly (Supplementary Table S2), suggesting that there is no apparent necessity to produce them once the biofilm is established, due to the potential immune response by the plant. Accordingly, genes involved in plant immune response are not activated (Supplementary Table S3). A similar situation is observed during formation of the legume plant-rhizobium interaction when LPS of rhizobia undergo structural modifications, and one of the explanations for this event is minimization of the plant immune response to bacterial invasion^62^,^63^,^64^.

In response to biofilm formation by *Burkholderia* Q208, roots expand aerenchyma as a likely response to low oxygen concentrations due to the combined effects of the physical impediment of the biofilm to oxygen diffusion into roots and high oxygen consumption by bacteria. In legume nodules, oxygen is absorbed with high affinity by leghaemoglobin to create the microaerobic environment conducive to BNF *via* the nitrogenase complex^65^. Effective compartmentalization in root nodules restricts low concentration of free oxygen to nodules and maintains the aerobic environment for roots tissues of the legumes. It may be less straightforward to achieve this situation in sugarcane, where the bacteria occur in a less-compartmentalised (although microaerobic) biofilm thus reducing oxygen concentration in the roots. Increased thickness and volume may allow sugarcane roots to be less affected by the microaerobic conditions at their surface, while the expanded aerenchyma facilitates increased air diffusion into roots. An elevated level of haemoglobin as deduced by the over-expression of the haemoglobin ortholog *AHB1/GLB1* in sugarcane roots presumably also contributes to increased oxygen delivery to roots. Together, these adaptations allow roots to remain aerobic while also enabling the conditions essential for BNF.

The association between *Burkholderia* Q208 and plants requires a significant amount of energy to support bacterial growth and function, including the energy-demanding process of BNF. Indeed, in the rhizosphere, *Burkholderia* Q208 activate several pathways that favor energy production under both aerobic and microaerobic conditions (Fig. 6a). As a result, the bacteria experience an energy-rich, oxygen-depleted environment, two features required for BNF (Fig. 6b). Our results suggest that over-expression of cytochrome *bd* and F0/F1 ATP synthase contributes to both aspects: firstly, it is responsible for increased production energy in aerobic and probably sub-aerobic conditions. Indeed, cytochrome *bd* has a very high affinity for O_2_, which implies that it is able to function at low oxygen concentration. Secondly, it is the factor that triggers the depletion of oxygen in *Burkholderia* cells, creating a microaerobic environment essential for BNF, as shown in *Azotobacter vinelandii*^41^ and *Klebsiella pneumonia*^66^.

However, by inducing anoxic conditions the *Burkholderia* Q208 potentially create adverse conditions for aerobic respiration: under low oxygen conditions, aerobic metabolism leads to accumulation of NAD(P)H which inhibits citrate synthase and isocitrate dehydrogenase, thereby impeding the Krebs cycle^67^. *Burkholderia* Q208 is an obligate aerobe and therefore lacks fermentation pathways that would restore regular cell redox potential, thus it is crucial for the bacterium to have an alternative system for oxidizing of NADH. The adaptation of *Burkholderia* consists in storing the surplus of carbon and energy in the form of PHB granules (Supplementary Figure S3). PHB metabolism is tightly linked to the redox state of the cell, and its accumulation in diazotrophs such as *Azotobacter* and *Rhizobium* is induced by oxygen limitation^67^. In *Azotobacter*, PHB serves as an alternative electron acceptor, relieving inhibition of the Krebs cycle by increasing the cells’ redox potential^67^.

Besides regulating the redox state of the cell, PHB provides a competitive advantage by allowing storage and mobilization of carbon in response to environmental changes. Upregulation of the genes involved in PHB degradation in *Burkholderia* Q208 indicates that PHB granules are used. The data suggest that under microaerobic conditions, PHB mobilization serves the oxalate catabolism, as the up-regulated oxalate catabolic gene cluster includes the PHB depolymerase and the acetyl-CoA:oxalate-CoA transferase (Supplementary Table S5) which activates oxalate via transfer of CoA. Oxalotrophy is widespread in *Burkholderia* of the environmental clade^68^ which includes *Burkholderia* Q208, although uncommon elsewhere, and may provide additional selective advantage for establishing the association with sugarcane. Notably, *Burkholderia* Q208 possesses a further adaptation to microaerobic conditions in the form of the arginine-deiminase pathway which is also up-regulated in the biofilm.

Another important feature of *Burkholderia* Q208 is the activation of the C4-dicarboxylate transport (Dct) system. In the *Rhizobium*-legume symbiosis, bacteria rely strongly on carbon sources generated by the host^69^. Dct involves transport into bacterial cells of macromolecules such as succinate, malate, fumarate, and aspartate, which are metabolized under aerobic or anaerobic conditions^70^. Importantly, this transport system is essential in rhizobia-legume symbiosis as it is involved in transferring the dicarboxylates produced by the host plant to the bacteroids as a source of carbon and energy^71^.

Our data show that plant nitrogen acquisition is stimulated in the association, with significantly higher nitrogen concentration in root and shoot tissues and increased plant growth (Fig. 5b). In contrast, concentration of carbon in shoots remains steady and this suggests that carbon is not specifically supplied by bacteria, but rather is the result of photosynthesis. Strongly upregulated *dct* transport and PHB accumulation systems suggest that bacteria receive in return plentiful and perhaps excessive amounts of plant photosynthates.

In summary, we provide multiple lines of evidence that the mutually beneficial association between *Burkholderia* Q208 and sugarcane brings about substantial molecular and morphological adaptations in both partners. This study reveals new aspects of the interactions between roots and bacteria occurring in the plant rhizosphere.

## Additional Information

**How to cite this article**: Paungfoo-Lonhienne, C. *et al*. Crosstalk between sugarcane and a plant-growth promoting *Burkholderia* species. *Sci. Rep.* **6**, 37389; doi: 10.1038/srep37389 (2016).

**Publisher’s note:** Springer Nature remains neutral with regard to jurisdictional claims in published maps and institutional affiliations.

## Supplementary Material

Supplementary Information

## Figures and Tables

**Table 1:** Mapping results of RNA-Seq reads.

Sample^a^	BK	P	PBK	
Total reads	32,924,254	225,281,182	225,405,310	
After trimming	31,215,516	215,930,334	216,240,114	
Reference genome	*Burkholderia*	Sorghum	*Burkholderia*	Sorghum
Mapped reads	30,647,488	98,978,000	13,130,108	86,768,000
Unmapped	568,028	116,952,334	116,342,006	

^a^Description: BK, *Burkholderia* transcriptome; P, sugarcane transcriptome; PBK, mixed transcriptome.
