# Supplementary material for: Crosstalk between sugarcane and a plant-growth promoting Burkholderia species
Source: Sci Rep. 2016 Nov 21;6:37389. doi: 10.1038/srep37389 (PMC5116747; doi:10.1038/srep37389)
Supplement: Supplementary Information [file srep37389-s1.pdf]

Supplementary information

Crosstalk between sugarcane and a plant-growth promoting *Burkholderia* species

Chanyarat Paungfoo-Lonhienne, Thierry G. A. Lonhienne, Yun Kit Yeoh, Bogdan C. Donose, Richard I. Webb, Jeremy Parsons, Webber Liao, Evgeny Sagulenko, Prakash Lakshmanan, Philip Hugenholtz, Susanne Schmidt, Mark A. Ragan

Supplementary Table S1. Counts of mapped reads of *Burkholderia* Q208 transcripts involved in biofilm biosynthesis and quorum sensing.

Gene ID	BK	PBK	Fold change	Gene function
Clusters bce-I and bce-II				
PROKKA-1.7.2_MINCED_AGP.PL_01760	561	950	1.7	acyltransferase 3 (bceS)
PROKKA-1.7.2_MINCED_AGP.PL_01761	96	1035	10.8	group 1 glycosyl transferase (bceT)
PROKKA-1.7.2_MINCED_AGP.PL_01762	34	311	9.3	polysaccharide biosynthesis protein (bceR)
PROKKA-1.7.2_MINCED_AGP.PL_01763	86	851	9.8	NHL repeat-containing protein (bceQ)
PROKKA-1.7.2_MINCED_AGP.PL_01764	11	38	3.5	hypothetical protein (bceP)
PROKKA-1.7.2_MINCED_AGP.PL_01765	23	270	11.9	GDP-mannose, C6-dehydratase (bceO)
PROKKA-1.7.2_MINCED_AGP.PL_01766	49	307	6.3	Fis family transcriptional regulator (bceN)
PROKKA-1.7.2_MINCED_AGP.PL_01767	42	527	12.6	group 1 glycosyl transferase (bceK)
PROKKA-1.7.2_MINCED_AGP.PL_01768	38	433	11.3	group 1 glycosyl transferase (bceJ)
PROKKA-1.7.2_MINCED_AGP.PL_01769	60	436	7.2	transmembrane protein (bceI)
PROKKA-1.7.2_MINCED_AGP.PL_01770	46	483	10.5	group 1 glycosyl transferase (bceH)
PROKKA-1.7.2_MINCED_AGP.PL_01771	62	529	8.5	family 2 glycosyl transferase (bceG)
PROKKA-1.7.2_MINCED_AGP.PL_01772	134	1350	10.1	exopolysaccharide tyrosine-protein kinase (bceF)
PROKKA-1.7.2_MINCED_AGP.PL_01773	76	705	9.3	polysaccharide export protein (bceE)
PROKKA-1.7.2_MINCED_AGP.PL_01774	81	646	7.9	protein tyrosine phosphatase (bceD)
PROKKA-1.7.2_MINCED_AGP.PL_01775	202	2961	14.6	UDP-glucose dehydrogenase (bceC)
PROKKA-1.7.2_MINCED_AGP.PL_01776	213	1735	8.1	undecaprenyl-phosphate glucose phosphotransferase (bceB)
PROKKA-1.7.2_MINCED_AGP.PL_01777	706	5191	7.4	mannose-1-phosphate guanylyltransferase (bceA)
Quorum sensing (BraI/R)				
PROKKA-1.7.2_MINCED_AGP.PL_04008	34219	15433	0.5	LuxR family transcriptional regulator (braR)
PROKKA-1.7.2_MINCED_AGP.PL_04009	44817	15978	0.4	hypothetical protein (rsaI)
PROKKA-1.7.2_MINCED_AGP.PL_04010	667	1328	2.0	Acyl-homoserine-lactone synthase (braI)

Fold enrichment denotes the transcription of genes in PBK compared with BK.

The bacterium were grown separately on MS medium (BK) or in association with sugarcane (PBK).

Supplementary Table S2. Counts of mapped reads *of Burkholderia* Q208 transcripts involved in lipopolysaccharide biosynthesis.

Gene ID	BK	PBK	Fold change	Gene function
Cluster 1				
PROKKA-1.7.2_MINCED_AGP.PL_02239	33	6	0.18	GtrA family protein
PROKKA-1.7.2_MINCED_AGP.PL_02240	298	171	0.57	hypothetical protein
PROKKA-1.7.2_MINCED_AGP.PL_02241	1268	267	0.21	O-antigen polymerase
PROKKA-1.7.2_MINCED_AGP.PL_02242	2679	388	0.14	group 1 glycosyl transferase
PROKKA-1.7.2_MINCED_AGP.PL_02243	274	57	0.21	glycosyl transferase family protein
PROKKA-1.7.2_MINCED_AGP.PL_02244	217	189	0.87	lipopolysaccharide heptosyltransferase I (p value > 0.05))
PROKKA-1.7.2_MINCED_AGP.PL_02245	145	42	0.29	methyl-accepting chemotaxis sensory transducer
PROKKA-1.7.2_MINCED_AGP.PL_02246	295	123	0.42	glycosyl transferase family 4
PROKKA-1.7.2_MINCED_AGP.PL_02247	803	597	0.74	glycosyl transferase family protein
PROKKA-1.7.2_MINCED_AGP.PL_02248	451	329	0.73	NAD-dependent epimerase/dehydratase
PROKKA-1.7.2_MINCED_AGP.PL_02249	1881	859	0.46	polysaccharide biosynthesis protein CapD
PROKKA-1.7.2_MINCED_AGP.PL_02250	451	354	0.79	NAD-dependent epimerase/dehydratase
PROKKA-1.7.2_MINCED_AGP.PL_02251	645	190	0.29	GDP-mannose 4,6-dehydratase
PROKKA-1.7.2_MINCED_AGP.PL_02252	65	39	0.60	glycosyl transferase family protein
PROKKA-1.7.2_MINCED_AGP.PL_02253	3817	1118	0.29	sugar acetylase
PROKKA-1.7.2_MINCED_AGP.PL_02254	507	612	1.21	glycosyl transferase family protein
PROKKA-1.7.2_MINCED_AGP.PL_02255	579	420	0.72	ABC transporter-like protein
PROKKA-1.7.2_MINCED_AGP.PL_02256	640	301	0.47	polysaccharide ABC efflux pump, inner membrane subunit
Cluster 2				
PROKKA-1.7.2_MINCED_AGP.PL_03978	32	11	0.35	glycosyl transferase group 1
PROKKA-1.7.2_MINCED_AGP.PL_03979	84	35	0.42	exopolysaccharide transport protein family protein
PROKKA-1.7.2_MINCED_AGP.PL_03980	11	3	0.24	protein tyrosine phosphatase
PROKKA-1.7.2_MINCED_AGP.PL_03981	58	7	0.12	polysaccharide export protein
PROKKA-1.7.2_MINCED_AGP.PL_03982	23	10	0.42	hypothetical protein
PROKKA-1.7.2_MINCED_AGP.PL_03983	23	7	0.31	UTP-glucose-1-phosphate uridylyltransferase
PROKKA-1.7.2_MINCED_AGP.PL_03984	32	14	0.42	acyltransferase 3
PROKKA-1.7.2_MINCED_AGP.PL_03985	60	27	0.45	hypothetical protein
PROKKA-1.7.2_MINCED_AGP.PL_03986	67	10	0.16	AraC family transcriptional regulator
PROKKA-1.7.2_MINCED_AGP.PL_03987	70	32	0.45	AraC family transcriptional regulator
PROKKA-1.7.2_MINCED_AGP.PL_03988	233	16	0.07	flagellar transcriptional activator FlhD
PROKKA-1.7.2_MINCED_AGP.PL_03989	117	57	0.48	sugar transferase
PROKKA-1.7.2_MINCED_AGP.PL_02001	142	53	0.37	Lipid A-core-O-antigen ligase

Fold enrichment denotes the transcription of genes in PBK compared with BK.
Bacteria were grown separately on MS medium (BK) or in association with sugarcane (PBK).

Supplementary Table S3. Counts of mapped reads of sugarcane transcripts involved in immune response.

Gene ID	P	PBK	Fold change	Gene function
Sb04g000530	363	203	0.56	Ortholog NOA1 (NO ASSOCIATED 1)/ nitric-oxide synthase
Sb06g028760	39	87	2.23	Ortholog FLS2 (FLAGELLIN-SENSITIVE 2)
Sb04g023810	2047	1280	0.63	Ortholog of BAK1
Sb01g040360	990	1347	1.36	Ortholog MEKK1 (MAP KINASE KINASE 1)
Sb10g003310	1374	941	0.68	Ortholog ATMKK2 (MAP KINASE KINASE 2)
Sb07g014650	964	839	0.87	Ortholog ATMKK3 (MITOGEN-ACTIVATED PROTEIN KINASE KINASE 3)
Sb10g006080	343	223	0.65	OrthologATMKK4 (MITOGEN-ACTIVATED PROTEIN KINASE KINASE 4)
Sb04g035370	278	533	1.92	Ortholog ATMKK5 (MITOGEN-ACTIVATED PROTEIN KINASE KINASE 5)
Sb05g008160	743	615	0.83	Ortholog ATMKK5 (MITOGEN-ACTIVATED PROTEIN KINASE KINASE 5)
Sb03g033190	685	1066	1.56	Ortholog MKK6 (MAP KINASE KINASE 6)

Fold enrichment denotes the transcription of genes in PBK compared with P.
The sugarcane were grown separately on MS medium (P) or in association with sugarcane (PBK).

Supplementary Table S4. Counts of mapped reads of *Burkholderia* Q208 transcripts involved in chemotaxis and flagella biosynthesis.

Gene ID	BK	PBK	Fold change	Gene function
PROKKA-1.7.2_MINCED_AGP.PL_06258	581	213	0.37	CheA signal transduction histidine kinase
PROKKA-1.7.2_MINCED_AGP.PL_06259	154	46	0.30	CheW protein
PROKKA-1.7.2_MINCED_AGP.PL_06260	242	97	0.40	methyl-accepting chemotaxis sensory transducer
PROKKA-1.7.2_MINCED_AGP.PL_06261	16	4	0.28	hypothetical protein
PROKKA-1.7.2_MINCED_AGP.PL_06262	112	57	0.51	CheR-type MCP methyltransferase
PROKKA-1.7.2_MINCED_AGP.PL_06263	87	44	0.51	chemoreceptor glutamine deamidase CheD
PROKKA-1.7.2_MINCED_AGP.PL_06264	102	67	0.66	chemotaxis-specific methylesterase
PROKKA-1.7.2_MINCED_AGP.PL_06265	31	20	0.66	chemotaxis protein CheY
PROKKA-1.7.2_MINCED_AGP.PL_06266	111	80	0.72	chemotaxis phosphatase CheZ
PROKKA-1.7.2_MINCED_AGP.PL_06268	1172	567	0.48	lipoprotein
PROKKA-1.7.2_MINCED_AGP.PL_06269	706	124	0.18	flagellar biosynthesis protein FlhB
PROKKA-1.7.2_MINCED_AGP.PL_06270	734	146	0.20	flagellar biosynthesis protein FlhA
PROKKA-1.7.2_MINCED_AGP.PL_06271	909	152	0.17	flagellar biosynthesis regulator FlhF
PROKKA-1.7.2_MINCED_AGP.PL_06272	427	83	0.19	flagellar biosynthesis protein FlhG
PROKKA-1.7.2_MINCED_AGP.PL_06273	406	87	0.22	RNA polymerase sigma-28 subunit FliA/WhiG
PROKKA-1.7.2_MINCED_AGP.PL_06274	251	142	0.57	hypothetical protein
PROKKA-1.7.2_MINCED_AGP.PL_06275	311	107	0.34	FlgN family protein
PROKKA-1.7.2_MINCED_AGP.PL_06276	425	178	0.42	Anti-sigma-28 factor FlgM family protein
PROKKA-1.7.2_MINCED_AGP.PL_06277	624	163	0.26	flagella basal body P-ring formation protein
PROKKA-1.7.2_MINCED_AGP.PL_06278	575	116	0.20	flagellar basal body rod protein FlgB
PROKKA-1.7.2_MINCED_AGP.PL_06279	736	92	0.12	flagellar basal body rod protein FlgC
PROKKA-1.7.2_MINCED_AGP.PL_06280	1191	227	0.19	flagellar basal body rod modification protein
PROKKA-1.7.2_MINCED_AGP.PL_06281	2398	458	0.19	flagellar hook protein FlgE
PROKKA-1.7.2_MINCED_AGP.PL_06282	1194	251	0.21	flagellar basal body rod protein FlgF
PROKKA-1.7.2_MINCED_AGP.PL_06283	1268	236	0.19	flagellar basal-body rod protein FlgG
PROKKA-1.7.2_MINCED_AGP.PL_06284	594	59	0.10	flagellar basal body L-ring protein
PROKKA-1.7.2_MINCED_AGP.PL_06285	560	150	0.27	flagellar basal body P-ring protein
PROKKA-1.7.2_MINCED_AGP.PL_06286	649	187	0.29	flagellar rod assembly protein/muramidase FlgJ

Gene ID	BK	PBK	Fold change	Gene function
PROKKA-1.7.2_MINCED_AGP.PL_06287	1434	1019	0.71	YcgR family protein
PROKKA-1.7.2_MINCED_AGP.PL_06288	1400	347	0.25	flagellar hook-associated protein FlgK
PROKKA-1.7.2_MINCED_AGP.PL_06289	797	214	0.27	flagellar hook-associated protein FlgL
PROKKA-1.7.2_MINCED_AGP.PL_06290	122	36	0.29	flagellar biosynthetic protein FliR
PROKKA-1.7.2_MINCED_AGP.PL_06291	40	4	0.09	flagellar biosynthetic protein FliQ
PROKKA-1.7.2_MINCED_AGP.PL_06292	124	40	0.32	flagellar biosynthesis protein FliP
PROKKA-1.7.2_MINCED_AGP.PL_06293	220	66	0.30	flagellar protein fliO
PROKKA-1.7.2_MINCED_AGP.PL_06294	251	65	0.26	flagellar motor switch protein FliN
PROKKA-1.7.2_MINCED_AGP.PL_06295	571	127	0.22	flagellar motor switch protein FliM
PROKKA-1.7.2_MINCED_AGP.PL_06296	298	51	0.17	flagellar basal body-associated protein FliL
PROKKA-1.7.2_MINCED_AGP.PL_06297	412	113	0.27	type-1 fimbrial protein subunit A
PROKKA-1.7.2_MINCED_AGP.PL_06298	891	170	0.19	flagellar hook-length control protein
PROKKA-1.7.2_MINCED_AGP.PL_06299	270	65	0.24	flagellar export protein FliJ
PROKKA-1.7.2_MINCED_AGP.PL_06300	484	99	0.20	ATPase FliI/YscN
PROKKA-1.7.2_MINCED_AGP.PL_06301	346	99	0.29	flagellar assembly protein H
PROKKA-1.7.2_MINCED_AGP.PL_06302	697	171	0.24	flagellar motor switch protein G
PROKKA-1.7.2_MINCED_AGP.PL_06303	566	169	0.30	flagellar MS-ring protein
PROKKA-1.7.2_MINCED_AGP.PL_06304	687	123	0.18	flagellar hook-basal body complex protein FliE
PROKKA-1.7.2_MINCED_AGP.PL_06305	334	41	0.12	flagellar protein FliS
PROKKA-1.7.2_MINCED_AGP.PL_06306	164	23	0.14	flagellar export chaperone
PROKKA-1.7.2_MINCED_AGP.PL_06307	300	71	0.24	Flagellar hook-length control protein-like protein
PROKKA-1.7.2_MINCED_AGP.PL_06308	70	31	0.44	flagellar biosynthetic protein FlhB

Fold enrichment denotes the transcription of genes in PBK compared with BK.
The bacterium were grown separately on MS medium (BK) or in association with sugarcane (PBK).

Supplementary Table S5. Counts of mapped reads of *Burkholderia* Q208 transcripts involved in energy pathway.

Gene ID	BK	PBK	Fold change	Gene function
Glycolysis				
PROKKA-1.7.2_MINCED_AGP.PL_05567	22	3518	159.1	1-phosphofructokinase (pfk)
PROKKA-1.7.2_MINCED_AGP.PL_05568	37	7628	203.9	phosphoenolpyruvate synthase (pps)
Acetyl-CoA synthesis				
PROKKA-1.7.2_MINCED_AGP.PL_01735	1295	3632	2.8	pyruvate dehydrogenase
Aerobic respiration				
Acetyl-CoA entry to Krebs cycle				
PROKKA-1.7.2_MINCED_AGP.PL_04980	695	2779	4.0	citrate synthase
Cytochrome bd-I complex (cluster)				
PROKKA-1.7.2_MINCED_AGP.PL_05777	468	16124	34.4	cytochrome bd-I ubiquinol oxidase, subunit I, CydA
PROKKA-1.7.2_MINCED_AGP.PL_05778	445	11982	26.9	cytochrome bd-I ubiquinol oxidase, subunit II, CydB
PROKKA-1.7.2_MINCED_AGP.PL_05779	144	3022	21.0	cyd operon protein YbgT, CydX
Cytochrome bd-II complex (cluster)				
PROKKA-1.7.2_MINCED_AGP.PL_05671	35	3896	111.9	cytochrome bd-II ubiquinol oxidase, subunit I, CbdA
PROKKA-1.7.2_MINCED_AGP.PL_05672	21	1010	49.0	cytochrome bd-II ubiquinol oxidase, subunit II, CbdB
Cytochrome bo (cluster)				
PROKKA-1.7.2_MINCED_AGP.PL_01563	313	305	1.0	cytochrome c oxidase subunit II
PROKKA-1.7.2_MINCED_AGP.PL_01564	452	377	0.8	cytochrome c oxidase subunit I
PROKKA-1.7.2_MINCED_AGP.PL_01565	149	124	0.8	cytochrome c oxidase subunit III
F0/F1 ATP synthase complex (cluster)				
PROKKA-1.7.2_MINCED_AGP.PL_05557	30	9934	326.3	F0F1 ATP synthase subunit beta
PROKKA-1.7.2_MINCED_AGP.PL_05558	4	1105	253.8	F0F1 ATP synthase subunit epsilon
PROKKA-1.7.2_MINCED_AGP.PL_05559	8	767	95.3	putative ATP synthesis-related protein
PROKKA-1.7.2_MINCED_AGP.PL_05560	7	580	77.9	F1/F0 ATPase, Methanosarcina type, subunit 2
PROKKA-1.7.2_MINCED_AGP.PL_05561	25	1281	51.3	F0F1 ATP synthase subunit A
PROKKA-1.7.2_MINCED_AGP.PL_05562	9	337	37.3	F0F1 ATP synthase subunit C
PROKKA-1.7.2_MINCED_AGP.PL_05563	17	1468	86.5	H+-transporting two-sector ATPase, B/B' subunit
PROKKA-1.7.2_MINCED_AGP.PL_05564	33	4043	123.5	F0F1 ATP synthase subunit alpha
PROKKA-1.7.2_MINCED_AGP.PL_05565	17	1552	89.1	H+-transporting two-sector ATPase, gamma subunit

Gene ID	BK	PBK	Fold change	Gene function
PHB-cycle				
PHB biosynthesis				
PROKKA-1.7.2_MINCED_AGP.PL_01872	3802	5485	1.4	acetyacetyl-CoA reductase (ortholog PhaB)
PROKKA-1.7.2_MINCED_AGP.PL_05532	13	9524	719.7	acetoacetyl-CoA reductase (otholog PhbB)
PROKKA-1.7.2_MINCED_AGP.PL_01874	3402	6098	1.8	PHB synthase (ortholog PhaC)
PROKKA-1.7.2_MINCED_AGP.PL_05531	57	13531	236.6	PHB polymerase domain-containing protein (ortholog PhbC)
PHB mobilization				
PROKKA-1.7.2_MINCED_AGP.PL_01661	171	9560	56.0	PHB depolymerase
PROKKA-1.7.2_MINCED_AGP.PL_01040	1875	4493	2.4	polyhydroxyalkanoate depolymerase (ortholog PhaZ)
PROKKA-1.7.2_MINCED_AGP.PL_05047	171	650	3.8	3-hydroxybutyrate dehydrogenase (hbd)
PROKKA-1.7.2_MINCED_AGP.PL_01072	1000	1412	1.4	acetoacetate–succinyl-CoA transferase
PROKKA-1.7.2_MINCED_AGP.PL_01073	584	944	1.6	acetoacetate–succinyl-CoA transferase
Anaerobic respiration				
Oxalotrophy pathway (cluster)				
PROKKA-1.7.2_MINCED_AGP.PL_01657	105	49883	474	formyl-coenzyme A transferase (Frc)
PROKKA-1.7.2_MINCED_AGP.PL_01658	84	29909	357	oxalyl-CoA decarboxylase (Oxc)
PROKKA-1.7.2_MINCED_AGP.PL_01659	28	15333	555	acetoacetyl-CoA:oxalyl-CoA transferase (ortholog YfdE)
PROKKA-1.7.2_MINCED_AGP.PL_01660	14	2772	203	putative membrane protein (3D homology with H+ symporter, membrane potential)
PROKKA-1.7.2_MINCED_AGP.PL_01661	171	9560	56	PHB depolymerase
PROKKA-1.7.2_MINCED_AGP.PL_03243	34	12	0.4	oxalate/formate antiporter (oxlT)
NAD-dependent formate dehydrogenase complex (reduction of NAD, aerobic))				
PROKKA-1.7.2_MINCED_AGP.PL_00528	1239	369	0.3	NAD-dependent formate dehydrogenase subunit delta
PROKKA-1.7.2_MINCED_AGP.PL_00529	11870	4280	0.4	NAD-dependent formate dehydrogenase subunit alpha
PROKKA-1.7.2_MINCED_AGP.PL_00530	4192	1392	0.3	NADH dehydrogenase (quinone)
PROKKA-1.7.2_MINCED_AGP.PL_00531	1542	547	0.4	NAD-dependent formate dehydrogenase subunit gamma
Arginine deiminase pathway (cluster)				
PROKKA-1.7.2_MINCED_AGP.PL_05525	43	11369	261.8	carbamate kinase (arcC)
PROKKA-1.7.2_MINCED_AGP.PL_05526	35	13220	374.0	ornithine carbamoyltransferase (arcB)
PROKKA-1.7.2_MINCED_AGP.PL_05527	36	15984	443.5	arginine deiminase (arcA)

Fold enrichment denotes the transcription of genes in PBK compared with BK.
The bacterium were grown separately on MS medium (BK) or in association with sugarcane (PBK).

SupplementaryTable S6. Counts of mapped reads of *Burkholderia* Q208 transcripts involved in DCT (Di-carboxylic transport).

Gene ID	BK	PBK	Fold change	Gene function
PROKKA-1.7.2_MINCED_AGP.PL_05825	317	433	1.4	Fis family transcriptional regulator (ortholog DctD)
PROKKA-1.7.2_MINCED_AGP.PL_05826	369	599	1.6	integral membrane sensor signal transduction histidine kinase (ortholog DctB)
PROKKA-1.7.2_MINCED_AGP.PL_05827	119	557	4.7	sodium:dicarboxylate symporter (ortholog DctA)

Supplementary Table S7. Counts of mapped reads of sugarcane transcripts involved in response to hypoxia.

Gene ID	P	PBK	Fold change	Gene function
Sb01g009450	34	155	4.6	ACS8; 1-aminocyclopropane-1-carboxylate synthase
Sb03g026000	414	4466	10.8	ACO1 (ACC OXIDASE 1); 1-aminocyclopropane-1-carboxylate oxidase
Sb01g042260	484	7873	16.3	AHB1 (ARABIDOPSIS HEMOGLOBIN 1); oxygen binding / oxygen transporter
Sb04g035160	3016	8684	2.9	APETALA2/ethylene response factor (RAP2.2); DNA binding / transcription factor
Sb02g005750	11	564	53.6	prolyl 4-hydroxylase alpha (role in hypoxia stress)

Supplementary Table S8. Counts of mapped reads of sugarcane transcripts involved in sucrose synthase.

Gene ID	P	PBK	Fold change	Gene function
Sb10g006330	51592	77374	1.5	Sucrose synthase 1
Sb01g033060	8510	26185	3	Sucrose synthase 2

For Supplementary Tables S6, S7, and S8:
Fold enrichment denotes the transcription of genes in PBK compared with P.
The sugarcane were grown separately on MS medium (P) or in association with sugarcane (PBK).

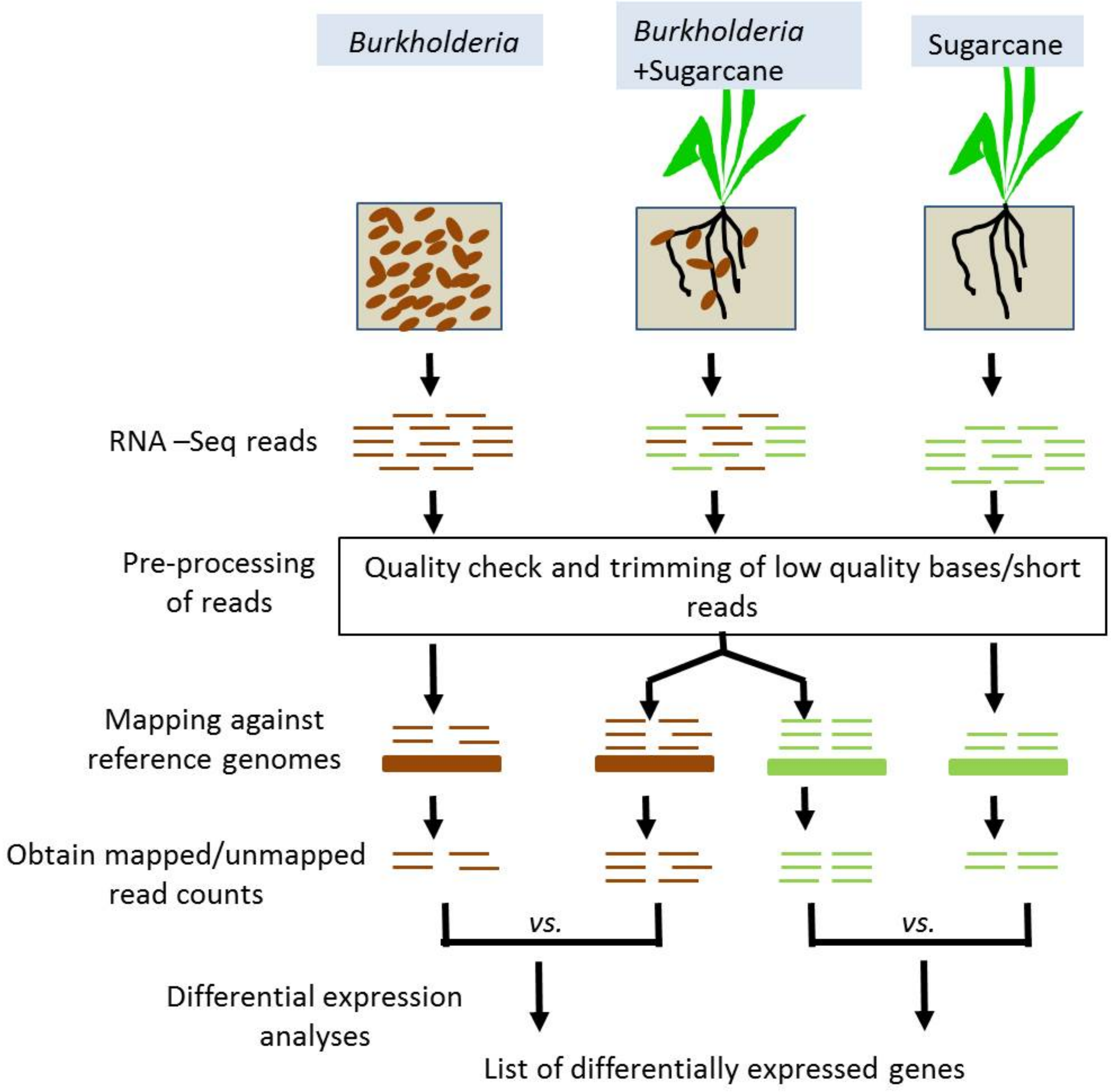

Supplementary Figure S1. Schematic representation of RNA-seq analysis of mixed transcriptome obtained from *Burkholderia*-inoculated sugarcane roots.

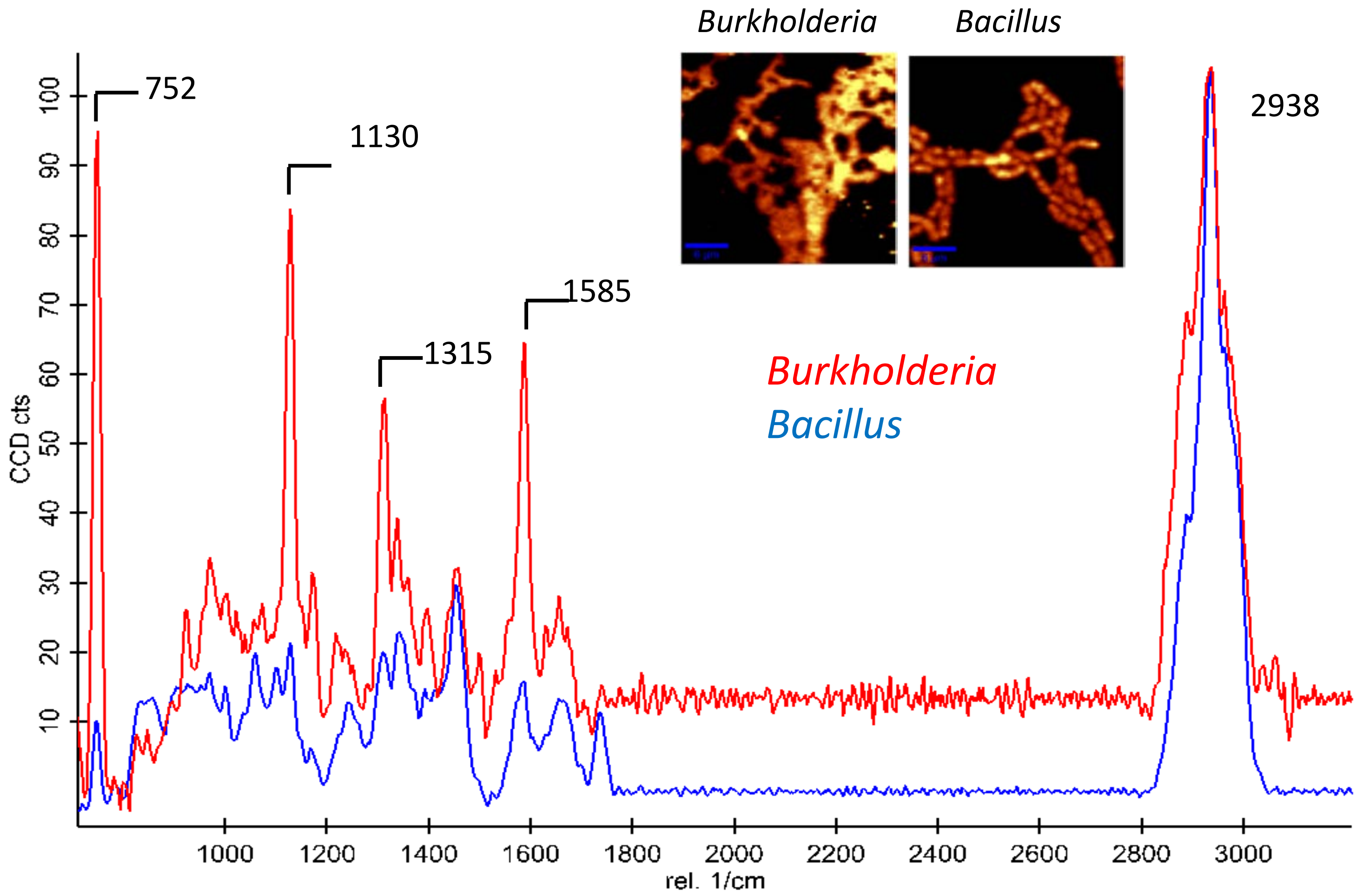

Supplementary Figure S2. Raman measurement of *Burkholderia australis* and *Bacillus megaterium* associated with sugarcane roots. Microscopic images (subset) taken with a 100x/NA0.9 air objective. Lighter colours represent greater signal intensity. Scale bars is 6 µm. Raman spectra acquired on *B. australis* (red) and *B. megaterium* (blue). Labelled peaks are the dominant resonant Raman band of Cytochromes. The Raman band at 2900 cm^{-1} represents CH stretching mode representing bacterial biomass.

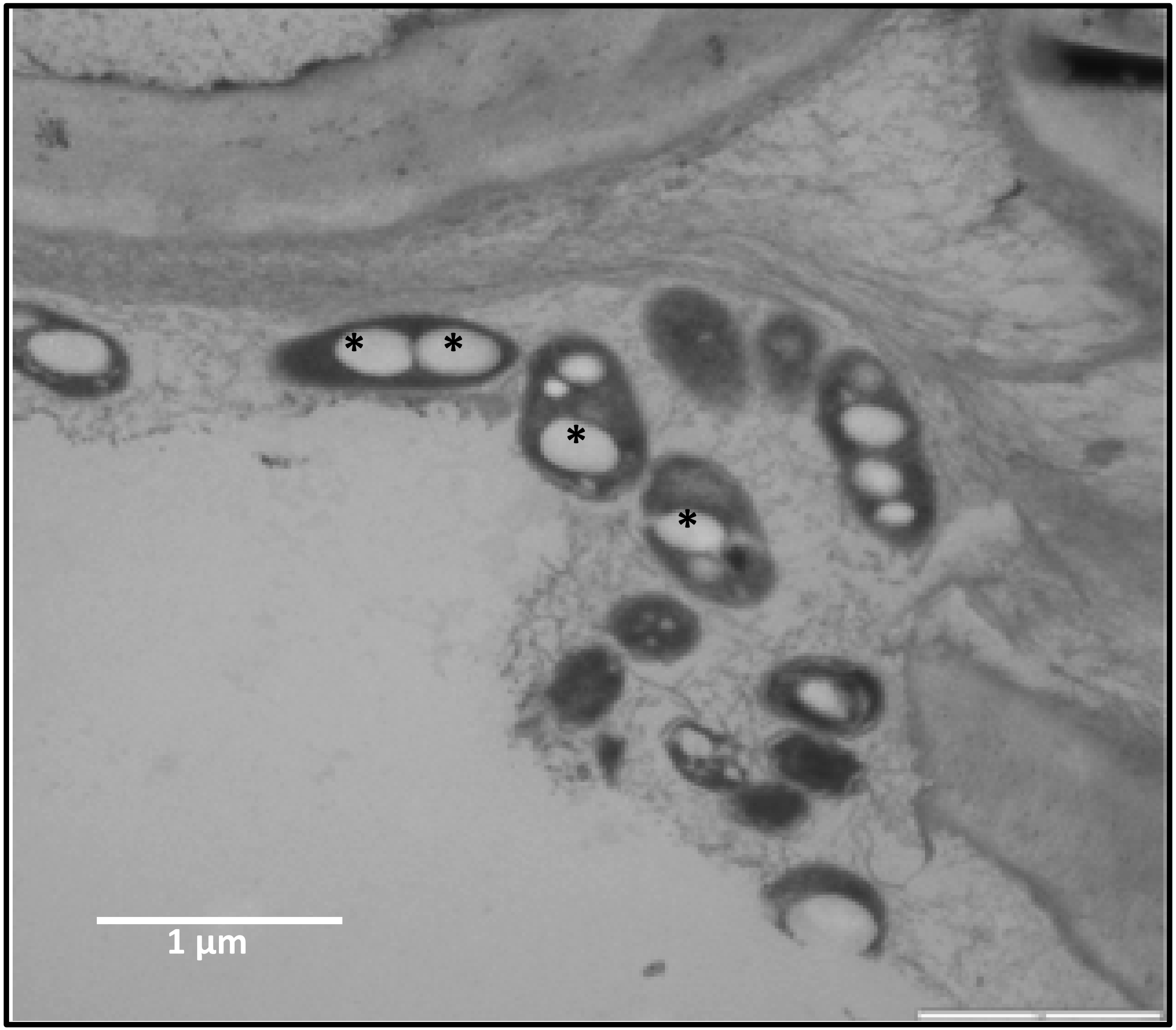

Supplementary Figure S3. Electron microscopy of *Burkholderia australis* associated with roots of sugarcane for 5 days. Poly-β-hydroxybutyrate (PHB) indicated by (*) was detected inside bacterial cells.

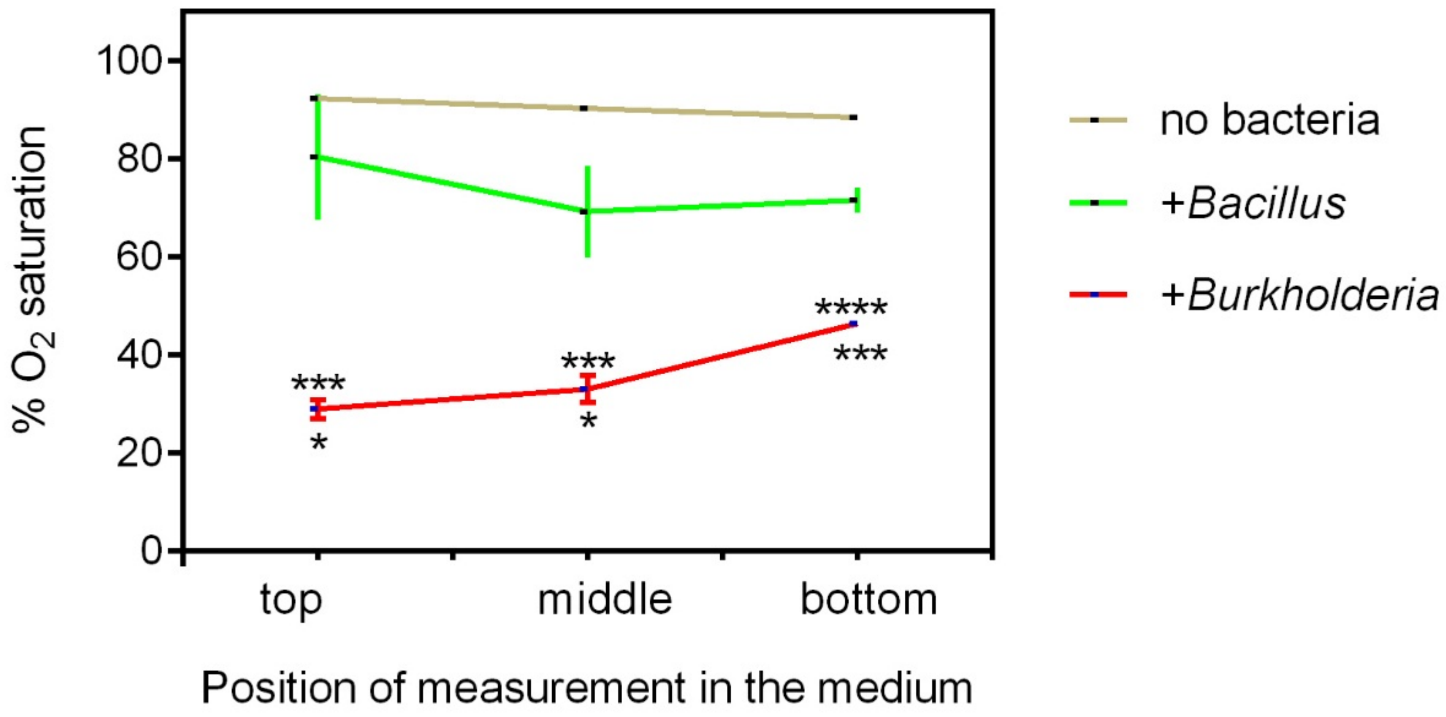

Supplementary Figure S4. Oxygen levels in the growth medium of 5-day-old cultures. The medium supporting the growth of plants treated with *B. australis* is oxygen-depleted compared to the medium of plants grown without *B. australis* or inoculated with *Bacillus megaterium* (control). Top, middle and bottom refers to the position in the growth vessels 9, 12, 15 cm from the vessel surface. Asterisks above and below bars indicate significant differences from the no bacteria and +*Bacillus* control, respectively: *, P<0.05; ***, P<0.001; ****, P<0.0001 (Student's *t* test).
